# Salinity effect on the metabolic pathway and microbial function in phenanthrene degradation by a halophilic consortium

**DOI:** 10.1186/s13568-018-0594-3

**Published:** 2018-04-25

**Authors:** Chongyang Wang, Yong Huang, Zuotao Zhang, Hui Wang

**Affiliations:** State Key Joint Laboratory of Environment Simulation and Pollution Control, School of Environment, Tsinghua University, Beijing, 100084 China

**Keywords:** Polycyclic aromatic hydrocarbons, Metabolic pathways, Halophilic consortium, *Marinobacter*, *Halomonas*, Community structure, C12O pathway, C23O pathway

## Abstract

With the close relationship between saline environments and industry, polycyclic aromatic hydrocarbons (PAHs) accumulate in saline/hypersaline environments. Therefore, PAHs degradation by halotolerant/halophilic bacteria has received increasing attention. In this study, the metabolic pathway of phenanthrene degradation by halophilic consortium CY-1 was first studied which showed a single upstream pathway initiated by dioxygenation at the C1 and C2 positions, and at several downstream pathways, including the catechol pathway, gentisic acid pathway and protocatechuic acid pathway. The effects of salinity on the community structure and expression of catabolic genes were further studied by a combination of high-throughput sequencing, catabolic gene clone library and real-time PCR. Pure cultures were also isolated from consortium CY-1 to investigate the contribution made by different microbes in the PAH-degrading process. *Marinobacter* is the dominant genus that contributed to the upstream degradation of phenanthrene especially in high salt content. Genus *Halomonas* made a great contribution in transforming intermediates in the subsequent degradation of catechol by using catechol 1,2-dioxygenase (C12O). Other microbes were predicted to be mediating bacteria that were able to utilize intermediates via different downstream pathways. Salinity was investigated to have negative effects on both microbial diversity and activity of consortium CY-1 and consortium CY-1 was found with a high degree of functional redundancy in saline environments.

**Electronic supplementary material:**

The online version of this article (10.1186/s13568-018-0594-3) contains supplementary material, which is available to authorized users.

## Introduction

Polycyclic aromatic hydrocarbons (PAHs) are wide spread petroleum pollutants that consist of two or more fused benzene rings (Haritash and Kaushik 2009). In recent years, because of the close association between saline/hypersaline environments and the petroleum industry, PAHs are found to accumulate in such environments (Debajyoti et al. 2016). Given the hyperosmosis and decreased bioavailability of PAHs, biodegradation in saline/hypersaline environments is quite difficult for traditional mesophilic microorganisms (Arulazhagan et al. 2014; Feng et al. 2012). Therefore, halotolerant and halophilic PAH-degrading microbes that show high activity in hypersaline environments have attracted increasing attention in recent years (Debajyoti et al. 2016).

Several halotolerant and halophilic microorganisms capable of degrading PAHs have been isolated from marine or other saline environments (Feng et al. 2012; Lin et al. 2014; Zhou et al. 2016), and their PAH-degrading pathways have been studied. For example, *Martelella* sp. AD-3 (Feng et al. 2012) isolated from saline soil has been proposed to mineralize phenanthrene by generating gentisic acid. Halophilic strain *Thalassospira* sp. SL5-1 (Zhou et al. 2016) has been identified to degrade pyrene through both gentisic acid and *o*-phthalic acid. However, little attention has been focused on the metabolic pathways in the halophilic PAH-degrading consortium (Dastgheib et al. 2012). As metabolic intermediates can be transferred among different microbes, the metabolic pathway of the PAH-degrading consortium is expected to be a more complex combination than pathways contained in pure cultures (Zafra et al. 2016; Zhao et al. 2016). Exploring the metabolic pathway of the halophilic PAH-degrading consortium is an important step in understanding the fate of PAHs in hypersaline environments (Arulazhagan and Vasudevan 2009; Debajyoti et al. 2016).

According to the studies of PAH-degrading pure cultures, PAHs can be degraded via several downstream pathways, such as catechol pathway, gentisic acid pathway and protocatechuic acid pathway. However, in the investigation of PAHs degradation by consortium, the downstream pathways had always been ignored. For example, Luan et al. detected the metabolic intermediates produced in the PAHs-degrading process by a bacterial consortium, suggesting the consortium contains multiple upstream pathways and one downstream pathway (Luan et al. 2006). Dastgheib et al. investigated the pathway of a halophilic PAH-degrading consortium with only one PAH-downstream pathway, namely catechol pathway (Dastgheib et al. 2012). The fluoranthene- and pyrene-degrading pathways of microbial consortiums have also been studied with massive intermediates and catabolic genes contained in PAH-upstream degrading process detected (Gupta et al. 2016; Zhao et al. 2016), however, only protocatechuic acid pathway have been detected in the PAHs downstream degrading process. In order to investigate the fate of PAHs in a consortium, it is very important to investigate how PAHs downstream pathways work in PAH-degradation process by consortiums (Moscoso et al. 2012), which will also give a deep vision of contribution made by different species during PAH-degrading process.

Identification of metabolites in downstream PAH-degrading pathways is often difficult as they are present in trace amounts (Luan et al. 2006). Therefore, the downstream PAH-degrading pathway must be studied by combining the detections of both metabolic intermediates and the genotypes and expression profiles of key metabolic genes involved in downstream PAH-degrading processes. Ring-hydroxylating dioxygenase (RHD), catechol 2,3-dioxygenase (C23O), catechol 1,2-dioxygenase (C12O), gentisate 1,2-dioxygenase (G12O), and protocatechuate 3,4-dioxygenase (P34O) are found to play pivotal roles during the PAHs biodegradation process (Azhari et al. 2007; Hesham et al. 2014; Táncsics et al. 2008; Wang 2007). Moreover, the present and expression of their encoding genes are measured to indicate the PAH-degrading potential (Mason et al. 2014; Paissé et al. 2012). Exploring the genotypes and expression of these genes can help to investigate the downstream pathways in a PAH-degrading consortium and the contribution made by different microbes in the PAH-degrading process.

In a previous study, a halophilic PAH-degrading consortium (CY-1) was successfully enriched under 10% salinity from petroleum contaminated soil from the Shengli oil field, which mainly consisted of genera *Marinobacter*, *Marispirillum*, and *Halomonas* (Wang et al. 2017). In this study, the metabolic pathway of consortium CY-1 was investigated by GC–MS. Several downstream pathways were found to coexist in the PAH-degrading process. We further analysed the effect of salinity on the community structure and expression of catabolic genes involved in these pathways by a combination of high-throughput sequencing, clone library construction, and real-time PCR of DNA and RNA. Moreover, further efforts were taken to isolate and identify the dominant species from this consortium to investigate the contribution made by the dominant genera to the PAH-degrading process in consortium CY-1. According to our knowledge, this is the first study on the metabolic pathways and effects of salinity on microbial function in halophilic PAH-degrading consortiums.

## Materials and methods

### Reagents

The standards of phenanthrene (98%), salicylic acid (99.5%), *o*-phthalic acid (99.8%), catechol (98%), protocatechuic acid (97%) gentisic acid (98%) and 1-hydroxy-2-naphthoic acid (97%) were purchased from Sinopharm Chemical Reagent Co., Ltd. (Shanghai, China). The DNAiso Reagent, Taq DNA polymerase, RNAiso Plus, DNase I, cDNA synthesis kit and SYBR^®^ Premix Dimer Eraser™ were purchased from TaKaRa (Dalian, China).

### Culture condition and phenanthrene degradation

The halophilic consortium CY-1, enriched from the contaminated soil from Shengli oil field (Wang et al. 2017), was cultured in sea salt-defined medium (SSDM) as previously described (Guo et al. 2016). This consortium was enriched and passed on NCBI database by Chongyang Wang (School of Environment, Tsinghua University, Beijing, China).

To study the salt effects on the phenanthrene degradation of CY-1, cell suspensions were added into 200 ml of SSDM with different salinity (3, 5, 10, and 20%), using phenanthrene as the sole carbon source with the initial concentration of 100 mg/l. And once after transferred into the same salinity, 3 ml samples were collected once or twice per day and then extracted using 5 ml of dichloromethane. Five flasks were used for one salinity treatment in parallel. The concentration of phenanthrene was detected using HPLC as previously described (Zhou et al. 2016).

### Detection of phenanthrene metabolic intermediates

To detect the intermediates produced by CY-1 during phenanthrene degradation, 100 ml cell suspensions were collected at day 2 and day 6. Each sample was extracted under different pH values (pH = 2, pH = 7 and pH = 12) using 50 ml dichloromethane one after the other. After extraction, these three extracts were mixed and concentrated using a rotatory evaporator and then dried under a stream of high purity nitrogen. Bis (trimethylsilyl) trifluoroacetamide (BSTFA and TMCS, volume ratio: 99/1) was used as the derivatization reagent so that active hydrogen atom(s) of the phenanthrene metabolites could be replaced by the trimethylsilyl (TMS) group (Si(CH3)3), *m/z* 73). For each standard compound (salicylic acid, 1-hydroxy-2-naphthoic acid, *o*-phthalic acid, protocatechuic acid, gentisic acid, catechol and phenanthrene), 0.1 mg was dissolved in 5 ml of dichloromethane and treated with the same process as the samples. Finally, the samples and the standard compounds were detected using GC–MS (Agilent 7890A GC, Inert MSD with TripleAxis Detector, Agilent, USA) as previously described (Zhou et al. 2016).

### DNA extraction, community structure detection and clone library construction

The genome DNA was extracted from consortium CY-1 cultured at 10% salinity and 3, 5, 20% salinities after transferred one times, using the DNeasy Blood & Tissue Kit (Qiagen, Germany) according to the manufacturer’s instructions. The 16S rRNA V3–V4 regions were further investigated using 454-pyrosequencing by the Sinobiocore Company (Beijing, China). The raw data had been submitted into NCBI database (accession number: SAMN07998561 to SAMN07998564).

The diversity of genes encoding RHD, C12O, C23O, G12O and P34O were investigated using a clone library according to the manufacturer’s instructions. The primers used for different function genes and their annealing temperatures are shown in Table 1, among which the primers for genes encoding RHD, C12O, C23O and P34O were used from previous studies (Ding et al. 2010; El et al. 2007; García et al. 2005; Sei et al. 1999). Approximately 100 clones were sequenced from each clone library, and the OTUs were divided at 90% similarity.

**Table 1:** Primers used in this study

Gene targets	Primers	Sequences	Amplicon length	Annealing temperatures °C	References
RHD	pahAc-f	5′-ATTGCGCTTAYCAYGGBTGG-3′	400	49	Ding et al. (2010)
	pahAc-r	5′-ATAGGTGTCTCCAACRAARTT-3′			
C12O	C12O-f	5′-ACCATCGARGGYCCSCTSTAY-3′	500	60	Sei et al. (1999)
	C12O-r	5′-GTTRATCTGGGTGGTSAG-3′			
C23O	C23O-f	5′-CGACCTGATCRSCATGACCGA-3′	280	50	García et al. (2005)
	C23O-r	5′-TYAGGTCAKMACGGTCA-3′			
G12O	G12O-f	5′-CGRTGATSTGGMTSGAYGG-3′	460	53	This study
	G12O-r	5′-CCAGCYVGGMACCACRAASA -3′			
P34O	P34O-f	5′-GCSCCSCTSGAGCCSAACTTC-3′	500	60	Azhari et al. (2007)
	P34O-r	5′-GCCGCGSAGSACGATRTCGAA-3′			
q-C12O	Q-C12f	5′-CCAGAGCGAATACAACATG-3′	174	50	This study
	Q-C12r	5′-GGAGACGAAGTAGTGGATG-3′			
q-C23O	Q-C23f	5′-TGTGGTGGTGACTACTTCT-3′	104	57	This study
	Q-C23r	5′-AAGCGTTCGTTCAGTACC-3′			
q-G12O	Q-Gf	5′-GAACAATCGCCAGGAAGT-3′	237	51	This study
	Q-Gr	5′-CATGGTAAGGATCGGTGAT-3′			
q-P34O	Q-Pf	5′-AGCACGGCTACTACCATT-3′	191	57	This study
	Q-Pr	5′-CGATACGCAGTTCACGAT-3′			

### Real-time PCR analyses of function genes in both DNA and RNA

To determine the copies of genes encoding C12O, C23O, G12O and P34O in total consortium DNA and their expression situations in phenanthrene degradation, DNA and RNA were extracted from samples that were collected once per day from culture growth under different salinities. DNA was extracted by the DNeasy Blood & Tissue Kit, and RNA was extracted by RNAiso according to the manufacturer’s instructions. The extracted RNA was reverse-transcribed by a cDNA synthesis kit according to the manufacturer’s instructions before conservation. Function genes and their transcripts were quantified in an iCycleriQ (Bio-Rad, USA) using the primer pairs shown in Table 1. Primers were designed according to the results obtained from the clone libraries. The standard curve for gene quantification was performed with the clones selected from each clone library. The gene expression level was calculated based on the transcript/gene ratio.

### Isolation and identification of pure cultures from CY-1

Several kinds of solid media were used to isolate more strains contained in CY-1. The media were all designed on the basis of SSDM containing 3% salinity and 1.5% agar (w/w). SSDM-Y was prepared using SSDM added with yeast extract (100 mg/l) under 3% salinity. SSDM-G was prepared using SSDM added with glucose (100 mg/l) with 3% salt content. SSDM-P was prepared using SSDM under 3% salinity directly. Each kind of plate was equally distributed with phenanthrene on the surface after coagulation. After being diluted 1000 times, 100 μl of diluted cell suspension was coated at the surface of each plate. After incubation under 30 °C for 4 days, each colony was picked using inoculating loop and then transferred to another plate by streaking for further purification and growth.

Taxonomic characteristics of each strain were analysed by 16S rRNA gene sequencing with primers 8F and 1492R, as shown in Table 1. Then, we also detected whether these isolates can degrade phenanthrene. Primers for genes encoding RHD, C12O, C23O, G12O and P34O were also used to detect whether these catabolic genes existed in these isolates.

### Accession numbers of sequences used in this study

All the cloned sequences in this study were submitted to NCBI database. The accession numbers of 16S rRNA cloned from the isolates were from MG386649 to MG386666. The accession numbers of the cloned catabolic genes were shown in Additional file 1: Table S1 and also shown in the related figures.

## Results

### Consortium structure and phenanthrene degradation

**Fig. 1:**
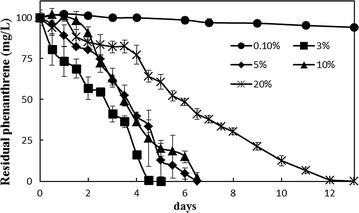
Effect of salinity on phenanthrene degradation by halophilic consortium CY-1. Consortium was cultivated batch-wise (second round) in SSDM under 0.1, 3, 5, 10 and 20% (w/w) salinity at 30 °C, 150 rpm. Samples were collected in the degradation process from day 1 to day 13

The halophilic bacterial consortium CY-1 was maintained by weekly transfers in SSDM with 10% salinity and phenanthrene (100 mg/l) for 3 months to become a stable microbial consortium. Phenanthrene degradation by consortium CY-1 was measured under different salinities. As shown in Fig. 1, no significant removal of phenanthrene occurs at 0.1% salinity, indicating that salt concentration was essential for the growth of functional microbes in CY-1. A salt content of 3% was considered as the most efficient salinity for CY-1, and the phenanthrene could be totally removed in 5 days. With the increased salt concentration, the phenanthrene removal rate was decreased. However, the removal rate showed no significant difference between salinities of 5 and 10% and the phenanthrene could be total removed in 6.5 days under these two salinities. The removal rate decreases quickly when the salinity increases to 20%, but the phenanthrene was still completely be degraded in 2 weeks.

**Fig. 2:**
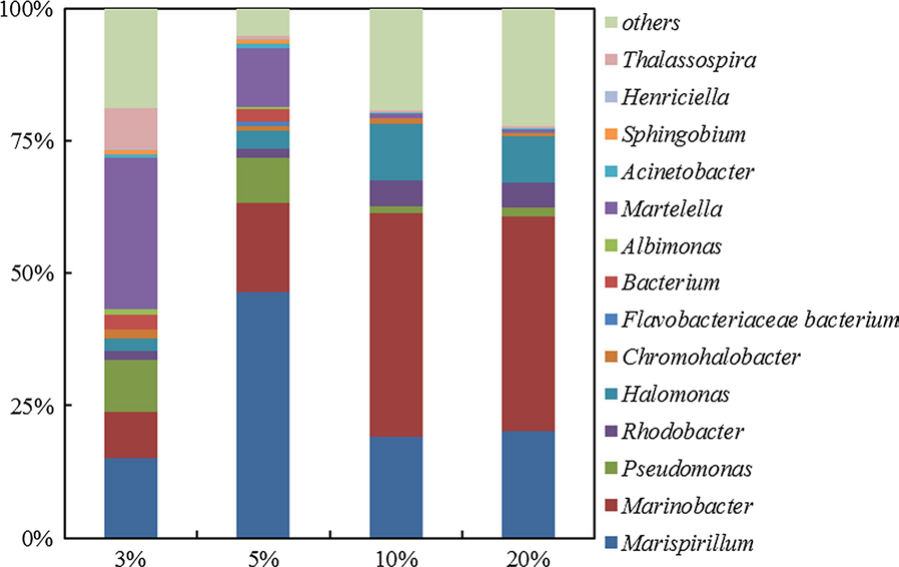
Community structure of CY-1 under different salinity. The genome DNA was extracted from samples collected from CY-1 cultured batch-wise (second round) under different salinities when 50% phenanthrene was removed. The sequence data were separated into different OTUs with 97% identity. The annotation of each OTU was identified using BLAST on NCBI website

The V3 and V4 hypervariable regions of the 16S rRNA gene were sequenced to analyse the community structure under different salinities. As shown in Fig. 2, *Marinobacter* and *Marispirillum* were recognized as the most dominant members in consortium CY-1, especially under high salinity. The abundances of *Marinobacter*, *Chromohalobacter Halomonas* and *Rhodobacter* were increased in the salt range of 3–20%, indicating that these bacteria had a great tolerance to high salinity. In contrast, the abundance of *Martelella*, which was predicted to be the dominant genus in CY-1 cultured under 3% salinity, decreased remarkably with the increase of salinity. The abundances of *Pseudomonas* and *Albimonas* were also decreased with increased salinity, suggesting a lower salinity tolerance of these genera. The microbial composition of consortium CY-1 showed no significant difference when cultured under 10 and 20% salinity, indicating that the community structure of consortium CY-1 was stable in hypersaline environments.

### Identification of metabolites produced in phenanthrene degradation

Seven metabolites, labeled metabolite 1–7 were detected in the phenanthrene-degrading process by CY-1 cultured at 10% salinity. These metabolites were all identified by comparison with the retention time (*Rt*) and mass spectra via the NIST library available in GC–MS and those detected from the authentic standards. As shown in Table 2, metabolite 1 was eluted at 35.79 min, with the major ions 178 (M+), 152, 89 and 26, which was considered as phenanthrene. Metabolite 2 was eluted at 39.31 min and had a molecular ion (M+) at *m/z* 332 and major fragment ions at *m/z* 317, 243, 185, 147 and 73, which was considered as TMS-derivatized 1-hydroxy-2-naphthoic acid. This metabolite was detected in phenanthrene degradation by the halophilic strain *Martelella* sp. AD-3 and was considered as a major accumulated intermediate in PAH metabolism (Feng et al. 2012). Metabolite 3 was eluted at 21.3 min, with the major ions 282 (M+), 267, 193, 178, 149, 91 and 73. This intermediate was considered as TMS-derivatized salicylic acid and was widely detected in phenanthrene degradation by bacteria such as halophilic strain *Thalassospira* sp. TSL5-1 (Zhou et al. 2016). Metabolite 4 was eluted at 22.95 min, which contained a molecular ion at *m/z* 310 and fragmentation ions at 295, 251, 221, 178, 103 and 73. This metabolite was identified as TMS-derivatized *o*-phthalic acid and was also detected in the phenanthrene-degrading process by *Thalassospira* sp. TSL5-1 (Zhou et al. 2016). Metabolite 5, with major ions 256 (M+), 239, 167, 136, and 73, was identified as TMS-derivatized catechol. Metabolite 6 and metabolite 7 were identified as TMS-derivatized gentisic acid and TMS-derivatized protocatechuic acid, respectively. Catechol, gentisic acid and protocatechuic acid were all key intermediates in different phenanthrene metabolic pathways and were the substrates for the ring-cleaving processes.

**Table 2:** Metabolites detected in phenanthrene degradation by consortium CY-1

Metabolite	Retention time	*m/z* of fragment ions (% relative intensity)	Identification
Metabolite 1	35.79	178 (178, 152, 89, 26)	Phenanthrene
Metabolite 2	39.31	336 (317, 243, 185, 147, 73)	1-Hydroxy-2-naphthoic acid
Metabolite 3	21.3	282 (267, 193, 178, 149, 91, 73)	Salicylic acid
Metabolite 4	22.95	310 (295, 251, 221, 178, 103, 73)	Phthalic acid
Metabolite 5	23.14	256 (254, 239, 167, 136, 73)	Catechol
Metabolite 6	35.54	370 (370, 311, 280, 223, 193, 128, 73)	Gentisic acid
Metabolite 7	34.54	369 (370, 296, 267, 252, 235, 177, 164, 150, 126, 73)	Protocatechuic acid

**Fig. 3:**
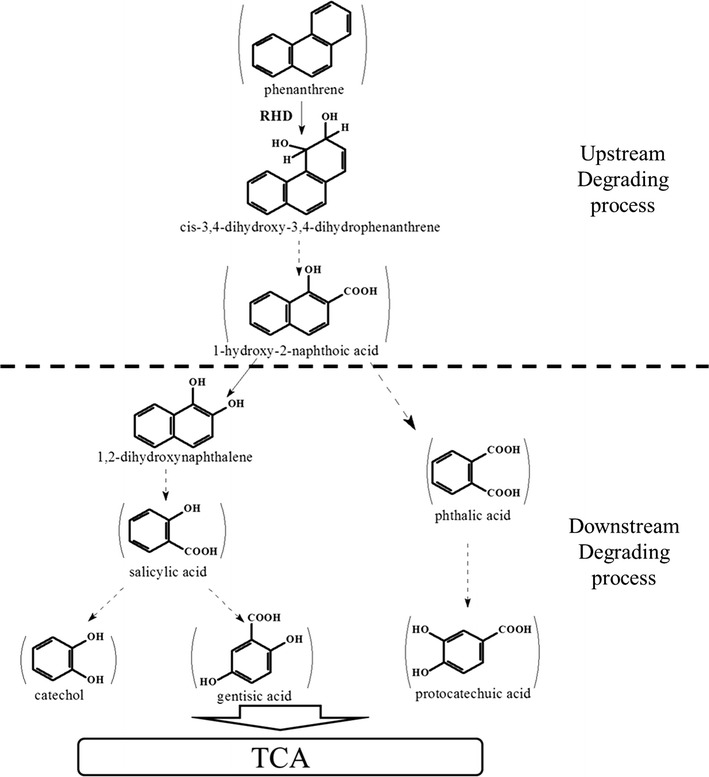
Proposed phenanthrene degradation pathway in the halophilic consortium CY-1. Compounds detected were shown in bound. Solid arrows indicate a single reaction while broken arrows represent two or more transformation steps

Based on the identified metabolites, namely, 1-hydroxy-2-naphthoic acid, salicylic acid, *o*-phthalic, catechol, gentisic acid and protocatechuic acid, and the pathways reported from PAH-degrading isolates, the phenanthrene metabolic pathway of CY-1 was predicted as shown in Fig. 3. Although neither dihydrodiols nor diols were found, the initial step of phenanthrene biodegradation by CY-1 was predicted to be dioxygenation at the 3,4-C positions because of the detection of 1-hydroxy-2-naphthoic acid, which was commonly found in phenanthrene biodegradation by *Pseudomonas* spp., *Sphingomonas* spp., and *Martelella* spp. (Balashova et al. 1999; Cho et al. 2006; Feng et al. 2012). The metabolic pathway was first separated from the produced 1-hydroxy-2-naphthoic acid by a direct ring cleavage or a decarboxylation process to form 1,2-dihydroxynaphthalene. The former could then form *o*-phthalic acid and protocatechuic acid to tricarboxylic acid (TCA) cycle. The latter could be further transformed to salicylic acid and then branched again by forming catechol or gentisic acid to TCA cycle.

### Clone libraries of metabolic genes in consortium CY-1

**Fig. 4:**
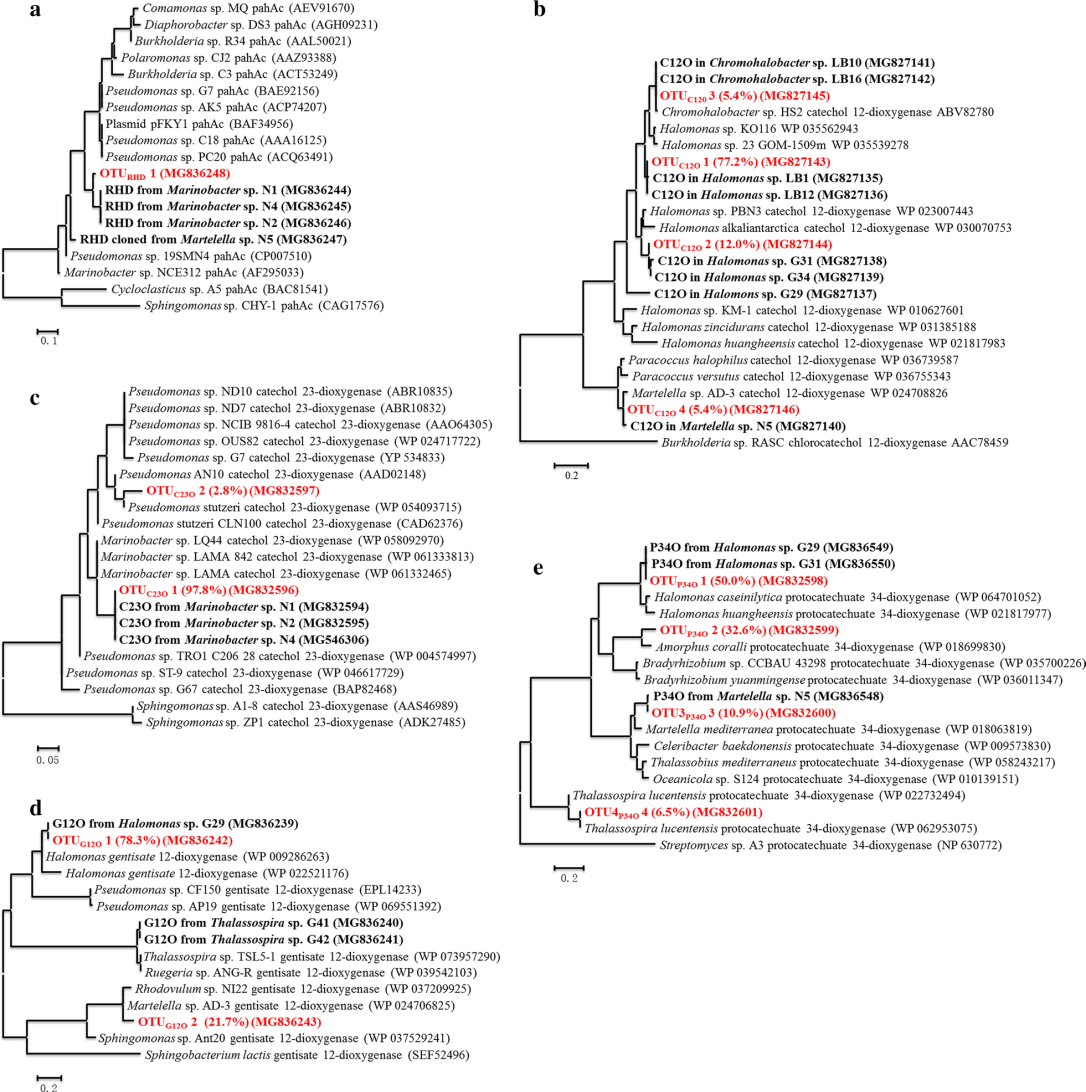
Phylogenetic trees of catabolic gene fragments obtained from clone libraries and isolated pure cultures. **a** Genes encoding RHDs; **b** genes encoding C12Os; **c** genes encoding C23Os; **d** genes encoding G12Os; **e** genes encoding P34O. The sequences obtained from clone libraries were separated into different OTUs with 90% identity. Each OTU was shown in red bold, with proportion shown in the brackets. The sequences of fragments obtained from isolated pure cultures were shown in black bold

C12O, C23O, G12O and P34O were the key enzymes involved in PAHs metabolism that participated in the complete cleavage of the aromatic ring of intermediate metabolites, namely, catechol, gentisic acid, and protocatechuic acid. Genes encoding these enzymes were usually used as biomarkers to indicate the PAH-degrading potential and downstream pathways. In this study, to further investigate the downstream pathways and the function of dominant populations in CY-1, clone libraries of genes encoding C12O, C23O, G12O, and P34O were constructed, and the results are shown in Fig. 4.

A total of 106 positive clones containing C23O genes were obtained in this study. The deduced amino acid sequences fell into two OTUs when 90% identity was used. OTU_C23O_ 1 with 103 clones was the dominant C23O gene group, which was closely related to the C23O genes contained in *Marinobacter*. The C23O gene in *Marinobacter* sp. LQ44 was found with 93% identity with OTU_C23O_ 1 cloned from CY-1. OTU_C23O_ 2 with 3 clones was found with 95% identity with the C23O genes contained in *Pseudomonas*.

A total of 92 positive clones containing C12O genes were obtained, and the deduced amino acid sequences fell into 4 OTUs when 90% identity was used. OTU_C12O_ 1 with 71 clones was most closely related to alleles from *Halomonas*, with 95% identity with *Halomonas pantelleriensis* (accession number: SDM34232) and *Halomonas korlensis* (accession number: SFU82093). OTU_C12O_ 2 with 11 clones was identified with 99% identity with *Halomonas taeanensis* (accession number: SDF94050), and 93% identified with *Halomonas* sp. PBN3 (accession number: WP_023007443). OTU_C12O_ 3 with 5 clones was closely related to C12O genes contained in *Chromohalobacter* sp. HS2 (accession number: ABV82780). OTU_C12O_ 4 with 5 clones was mostly related to the C12O genes in *Martelella* sp. AD-3 (97% identity, accession number: WP_024708826).

Clone libraries of G12O genes were constructed with 92 positive clones, and their deduced amino acid sequences fell into 2 OTUs belonging to *Halomonas* spp. (72 clones) and *Martelella* spp. (20 clones), respectively. The predicted amino acid sequence of OTU_G12O_ 1 showed identity with *Halomonas* sp. KO116 (accession number: WP_009286263) and *Halomonas* sp. 23_GOM-1509m N557DRAFT (accession number: WP_022521176). The predicted amino acid sequence of OTU_G12O_ 2 showed 94% identity with G12O genes contained in *Martelella* sp. AD-3 (accession number: WP_024706825).

Clone libraries of P34O genes with 92 positive clones were constructed. The deduced amino acid sequences were divided with 90% identity and fell into 4 OTUs. OTU_P34O_ 1 with 46 clones was found with 91% identity with *Halomonas caseinilytica* (accession number: WP_064701052) and 91% identity with *Halomonas elongata* (accession number: WP_041602201). OTU_P34O_ 2 with 30 sequences was identified with 88% identity with *Amorphous coralli* (accession number: WP_018699830). OTU_P34O_ 3 with 10 sequences was identified as closely related with the P34O genes contained in *Martelella mediterranea* (89% identity, accession number: WP_018063819); and OTU_P34O_ 4 with 6 sequences was mostly identified with *Thalassospira lucentensis* (100% identity, accession number: WP_062953075).

### Expression of catabolic genes

The RT-PCR reactions of genes encoding RHD, C12O, C23O, G12O and P34O were performed on both genomic DNA and RNA extracted from consortium CY-1 cultured under different salinities. As shown in Additional file 1: Figure S1, all these selected genes were induced in the PAH-degrading process, indicating that consortium CY-1 was able to completely mineralize phenanthrene at a wide range of salinities via multiple downstream pathways, namely, the C23O, C12O, G12O and P34O pathways. However, the expression levels of these catabolic genes were decreased with the increase in salinity, suggesting that high salinity can restrict the activity of microorganism even halophiles.

**Fig. 5:**
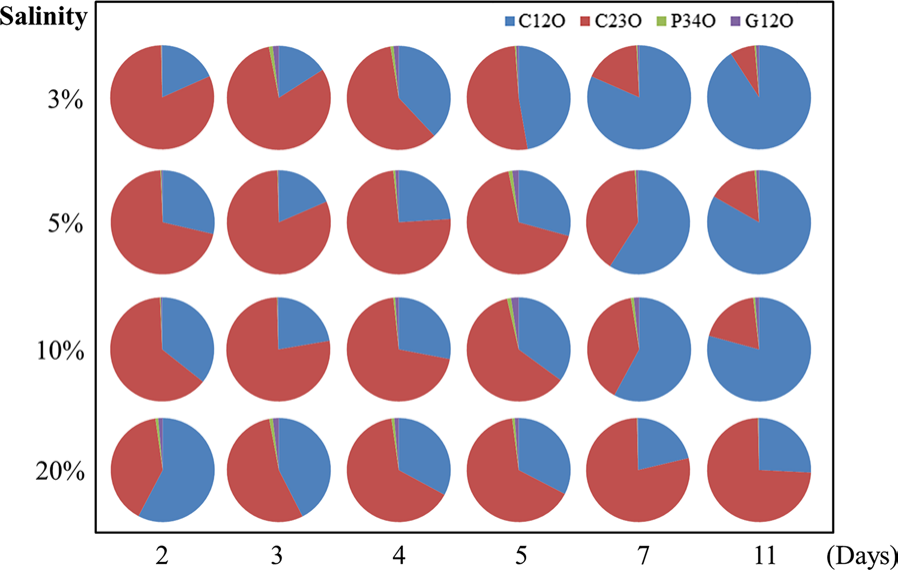
The ratio of genes encoding C12O, C23O, P34O and G12O in RNA extracted at different time of consortium CY-1 cultured under different salinities

To investigate which was the dominant pathway in the PAH-degrading process of consortium CY-1, the proportions of the quantity of genes encoding C12O, C23O, G12O and P34O in RNA were calculated. As shown in Fig. 5, genes encoding C12O and C23O showed a much higher proportion than genes encoding G12O and P34O, suggesting that the C12O and C23O pathways were the dominant downstream pathways in consortium CY-1. Although their encoding genes were induced in the PAH-degrading process, the G12O and P34O pathways were predicted to be less important. Interestingly, in the prophase of the PAH-degrading process, the proportion of genes encoding C23O was higher than genes encoding C12O. However, in the anaphase, the latter showed a higher proportion. This phenomenon suggests that, in consortium CY-1, different microbes contribute to different phases in the PAH-degrading process. Moreover, according to the result obtained from clone libraries (Fig. 4), *Marinobacter* was predicted to be the dominant genus in the prophase of the PAH-degrading process, and *Halomonas* was predicted to be a mediating bacterium that contributes to the anaphase of the PAH-degrading process, mainly via the C12O pathway.

### Pure culture isolation and identification

Bacteria in consortium CY-1 were isolated with SSDM-P, SSDM-G and SSDM-Y mediums. Each plate was coated with phenanthrene after solidification, and the colonies with clear zones were selected as potential phenanthrene-degrading bacteria. A total of 68 isolates were isolated, and finally, 18 strains (accession number: MG386649 to MG386666) with different 16S rRNA sequences were obtained. According to the 16S rRNA sequences, these isolates were identified as belonging to 7 genera, including four *Marinobacter* strains, five *Halomonas* strains, one *Martelella* strain, one *Marispirillum* strain, two *Chromohalobacter* strains, two *Thalassospira* strains and three *Alcanivorax* strains (Table 3). Primers used for the cloning of catabolic genes as described above, namely, genes encoding C12O, C23O, G12O, P34O and RHD, were also used to detect catabolic genes in these isolations as shown in Fig. 4.

**Table 3:** Identification of catebolic genes contained in species isolated form consortium CY-1

Strain	Isolation plate	Closest strains in Genbank (accession number)	Identity (%)	Calabolic genes encoding				
				RHD	C12O	C23O	G12O	P34O
N1	3%SSDM-P	*Marinobacter* sp. JXH-283 (KR012272)	97	√		√		
N2	3%SSDM-P	*Marinobacter* sp. CL9 (HM854280)	98	√		√		
N3	3%SSDM-P	*Marinobacter* sp. Xmb040 (HM854281)	99	√				
N4	3%SSDM-P	*Marinobacter* sp. JXH-283 (KR012272)	99	√		√		
N5	3%SSDM-P	*Martelella* sp. PETBA03 (JQ658408)	99	√	√			√
LB1	3%SSDM-Y	*Halomonas* sp. NIMMe9 (LC140966)	99		√			
LB2	3%SSDM-Y	*Halomonas* sp. USC33 (HQ441223)	99		√			
G29	3%SSDM-G	*Halomonas* sp. NIMMe9 (LC140966)	99		√		√	√
G31	3%SSDM-G	*Halomonas* sp. USC33 (HQ441223)	99		√			√
G34	3%SSDM-G	*Halomonas* sp. USC33 (HQ441223)	100		√			
LB15	3%SSDM-Y	*Marispirillum* sp. B142 (NR_044545)	97					
LB10	3%SSDM-Y	*Chromohalobacter* sp. L21-PYE-C7 (KJ187998)	99		√			
LB16	3%SSDM-Y	*Chromohalobacter* sp. JC125 (HE662816)	99		√			
G41	3%SSDM-G	*Thalassospira* sp. SW-3-3 (JX119044)	99				√	
G42	3%SSDM-G	*Thalassospira* sp. MCCC 1A02767 (EU440828)	99				√	
LB19	3%SSDM-Y	*Alcanivorax* sp. 2PR54-12 (EU440953)	99					√
LB20	3%SSDM-Y	*Alcanivorax* sp. NT N57 (AB166992)	99		√			
N10	3%SSDM-P	*Alcanivorax* sp. 2PR54-12 (EU440953)	100		√			

**Fig. 6:**
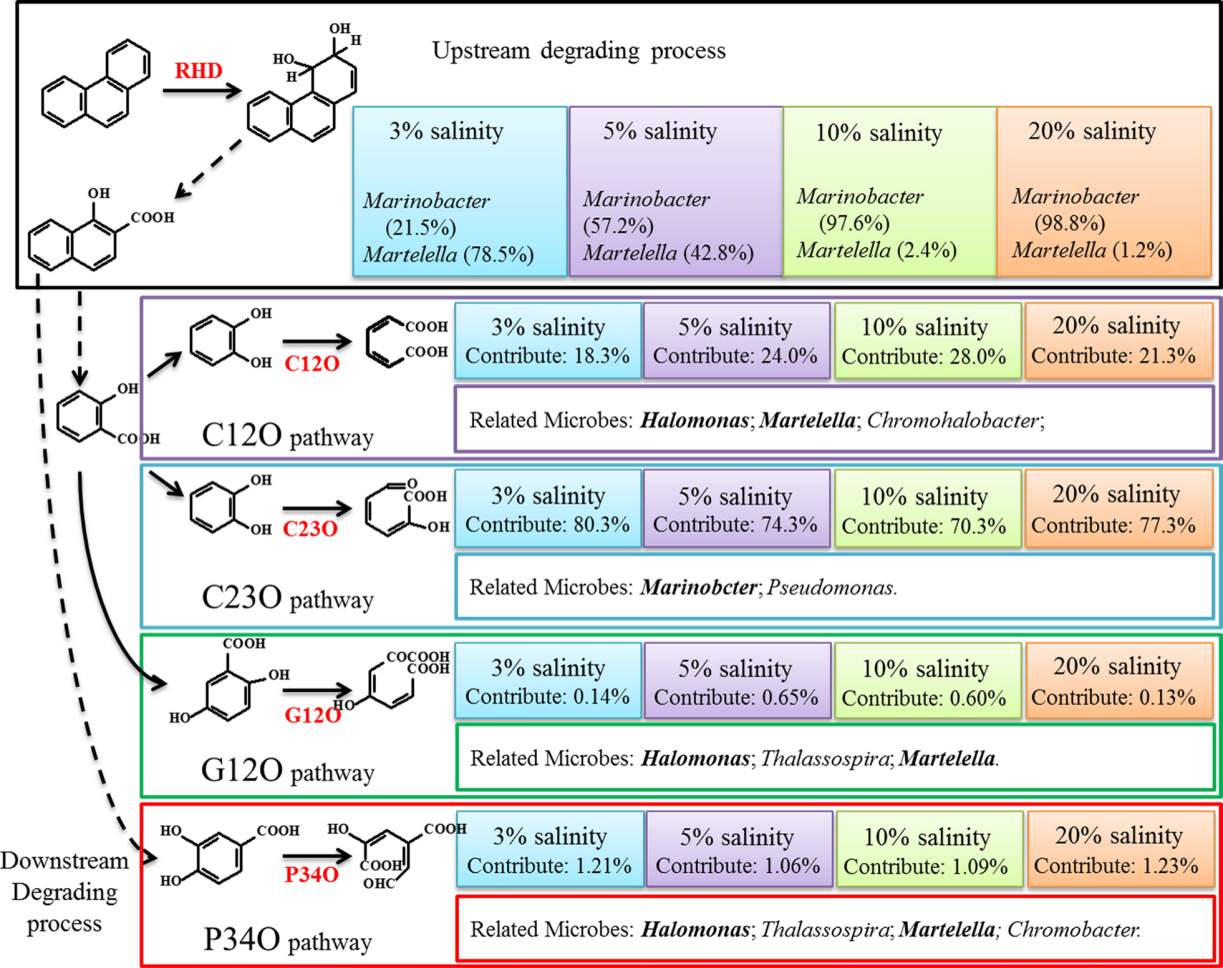
Contribution made by different microbes in consortium CY-1 when 50% phenanthrene was removed. The metabolic pathways were illustrated by the combination of the result obtained from intermediates detection and catabolic gene clone libraries. Solid arrow indicates a single reaction while broken arrow represents two or more transformation steps. Contribution made by *Marinobacter* and *Martelella* in the upstream degrading process was calculated by the abundance of these two genera in community structure. The contribution of each downstream pathway was calculated by the copies of each catabolic gene quantified from total RNA. The related microbes were summarized from the clone libraries and isolated pure cultures. Dominant genera in each downstream pathway which were determined by the proportion of catabolic gene in clone libraries and the abundance in community structure were shown in bold

*Marinobacter* strains (N1–N4) were all isolated from SSDM-P plates. Each of these isolates was found with a clear zone around the colony, indicating a potential phenanthrene-degrading ability. Using primers targeting different catabolic genes, all isolated *Marinobacter* species were detected with genes encoding RHD. Moreover, genes encoding C23O were also found in *Marinobacter* sp. N1, N2 and N4 (Table 3). These results indicated that *Marinobacter* was responsible for phenanthrene upstream degradation and took part in phenanthrene downstream degradation via the C23O pathway. The isolated *Martelella* (N5) was also found with a clear zone around the colony. Genes encoding RHD, C12O and P34O were cloned from this strain. Although isolated from SSDM-P plates, *Alcanivorax* sp. N10 was found with no clear zone around the colony and no ability to utilize phenanthrene, suggesting it may use agar as carbon source. Gene encoding C12O was detected in this strain. Other strain isolated from SSDM-G and SSDM-LB plates, namely *Halomonas*, *Chromohalobacter*, *Thalassospira*, and *Alcanivorax* strains, were also detected with no ability to directly utilize PAHs. However, different catabolic genes responsible for PAHs downstream degradation were found in these isolates, suggesting that they contributed to PAHs downstream degradation via different downstream pathways. *Marispirillum* sp. LB15 was isolated from SSDM-LB, which was detected with no catabolic genes. The predicted pathways of these bacteria were labelled on the proposed phenanthrene degrading pathway of consortium CY-1 (Fig. 6).

## Discussion

Although several bacterial isolates, such as *Pseudomonas* spp., *Sphingomonas* spp., and *Burkholderia* spp. (Haritash and Kaushik 2009; Waigi et al. 2015), were found to be able to completely mineralize PAHs by themselves, the cooperation among different bacterial species was the most important way to remove PAHs from the environment, which made it important to study PAHs biodegradation by bacteria mixtures, especially in hypersaline environments (Arulazhagan and Vasudevan 2009; Dastgheib et al. 2012; Zhao et al. 2016). Although PAHs degradation by microbe associations rather than by isolates was in accord with PAHs degradation in environments, reports are still limited regarding the metabolic mechanism of a halophilic phenanthrene-degrading consortium. As the produced metabolic intermediates could be used as substrates by other microbes, pathways of a PAH-degrading consortium were expected to be more complex than for PAH-degrading individuals. Based on the identification of metabolites, the metabolic pathway of consortium CY-1 was illustrated with a simple phenanthrene-degrading upstream pathway and several downstream pathways via catechol, gentisic acid and protocatechuic acid (Fig. 3). The produced catechol, gentisic acid and protocatechuic acid was further ring-cleaved by C12O, C23O, G12O, and P34O. The existence of various downstream pathways could help to rapidly transform intermediates and promote phenanthrene degradation by removing the toxicity generated by the accumulation of the produced intermediates (Gupta et al. 2016; Janbandhu and Fulekar 2011).

It was also important to understand the contribution made by different microbes and pathways contained in a PAH-degrading consortium. Using clone libraries of key catabolic genes contained in the phenanthrene metabolic map of consortium CY-1, the contribution of different species to phenanthrene degradation in consortium CY-1 was studied; this degradation was also identified by the isolation and identification of pure cultures. The metabolic pathways and contributions made by different microbes were postulated, as shown in Fig. 6. *Marinobacter* and *Martelella* were predicted to be responsible for the phenanthrene upstream degrading process. However, with the increase in salinity, *Marinobacter* was found to be more important than *Martelella*. According to the quantity of catabolic genes in total RNA, the dominant downstream pathway in consortium CY-1 was predicted to be the C23O pathway when 50% phenanthrene was degraded. Approximately 75% phenanthrene was predicted to be degraded through this pathway at this moment in all salinity conditions. However, this proportion decreased rapidly with the increase in degradation time. As shown in Fig. 5, the C12O pathway is predicted to be dominant in the anaphase of the phenanthrene-degrading process.

As shown in Fig. 2, consortium CY-1 was mainly composed of *Marinobacter*, *Marispirillum* and *Halomonas*, together with the genera of *Martelella*, *Pseudomonas*, *Thalassospira*, *Chromohalobacter*, etc. at 10% salinity. In a previous study (Wang et al. 2017), genes encoding RHDs in consortium CY-1are not conclusively shown to belong to *Marinobacter* strain (Fig. 3a). In this study, RHD genes were also detected in isolated *Martelella* spp. (Table 3), indicating that *Marinobacter* and *Martelella* together contributed to the phenanthrene upstream degradation process in CY-1. *Marinobacter* was first found by Hedlund et al. with an RHD closely related to *nah* dioxygenase (Hedlund et al. 2001). After the first discovery, *Marinobacter* was widely detected in PAH-polluted saline environments (Arulazhagan et al. 2014; Cui et al. 2008, 2014; Dastgheib et al. 2012; Vila et al. 2010), while the metabolic pathway of PAHs by *Marinobacter* was poorly reported. The *nah*-related PAH-degrading upstream gene cluster was once predicted to exist in *Marinobacter* species (Wang et al. 2017), indicating that *Marinobacter* may utilize phenanthrene via the same upstream pathway as *Pseudomonas* (Balashova et al. 1997), namely, the transformation from phenanthrene to 1-hydroxy-2-naphthoic acid, which was also detected in this study. The degradation of monoaromatic pollutants by *Marinobacter* has been widely studied (Berlendis et al. 2010; Dosta et al. 2011; Moxley and Schmidt 2012). *Marinobacter* sp. KM2 was identified as a phenol-utilizing bacterium that was able to mineralize phenol via the C23O pathway (Moxley and Schmidt 2012). The C23O gene was also located in the phenol-degrading gene cluster in the genome of *Marinobacter* sp. LQ44 (accession number: NZ_CP014754). In this study, genes encoding C23O were mainly investigated from *Marinobacter*, which was also induced in the prophase of the PAH-degrading process (Fig. 5) and detected in *Marinobacter* isolates (Table 3). Therefore, it was predicted that *Marinobacter* was the dominant genus to contribute to phenanthrene upstream degradation and to participate in phenanthrene downstream degradation via the C23O pathway in the prophase of the phenanthrene degrading process in CY-1.

The C12O pathway is also a dominant downstream pathway in consortium CY-1 that mainly originated from *Halomonas*. Especially in the anaphase of biodegradation, C12O genes were largely expressed and continued for a long time, suggesting that *Halomonas* made a large contribution to transforming the intermediates produced via phenanthrene degradation. Although it had been widely detected in the degradation of monoaromatic pollutants (Táncsics et al. 2008), this is the first time that the C12O pathway was identified as directly associated with PAHs degradation. Although detected with few copies, genes encoding G12O and P34O in *Halomonas* were found to be successfully induced during the phenanthrene degradation process, indicating that multiple enzymatic systems may coexist in *Halomonas*. These pathways were also identified in the aromatic hydrocarbon degradation process by *Halomonas* in previous studies (Castillo-Carvajal et al. 2014), such as *Halomonas campisalis* isolated by Celso et al.(Oie et al. 2007), which utilized benzoate via C12O pathway; *Halomonas* sp. IMPC isolated by Abdelkaf et al. was identified with the P34O pathway (Abdelkafi et al. 2006). A variety of pathways associated with aromatic hydrocarbons biodegradation were predicted to be the reason *Halomonas* widely discovered in PAHs contaminated marine environments (Castillo-Carvajal et al. 2014; Fathepure 2014; Yin et al. 2014).

*Marispirillum*, which was another dominant genus in consortium CY-1, was only once previously reported to be isolated from sea sediments (*Marispirillum* sp. B142) (Lai et al. 2009). In this study, the isolated strain *Marispirillum* sp. LB15 showed 97% identity with *Marispirillum* sp. B142. *Marispirillum* sp. LB15 was unable to grow in SSDM without a carbon source but was able to grow with the addition of yeast extract, indicating that *Marispirillum* was worked as a mediating bacterium in consortium CY-1. However, as no catabolic genes were detected in this strain, it was predicted that new genotypes existed in this genus. Other genera, such as *Thalassospira*, *Chromohalobacter* and *Alcanivorax* isolated from CY-1, were unable to utilize phenanthrene but were detected with some catabolic genes. It was predicted that these genera in consortium CY-1 were working as mediating microbes that contributed to the phenanthrene downstream degradation via multiple pathways. The utilization of the produced intermediates by mediating bacteria via different downstream pathways was predicted to be the reason the PAH-degrading rate by consortium CY-1 was much faster than isolated *Marinobacter* pure cultures (data not shown).

Several studies have reported the effect of salinity on the microbial community structure and enzyme activity in enriched microbial consortia (Fang et al. 2016; Guo et al. 2016; Jing et al. 2010) or the environment (Chen et al. 2017; Jing et al. 2010). The community structure of consortium CY-1 showed significant differences between culture conditions of high and low salinities (Fig. 2). The Shannon (H’) diversity indices of microbial composition when consortium CY-1 cultured under different salinities was calculated to evaluate the effect of salinity on the microbial diversity of consortium CY-1. The H’ value of consortium CY-1 cultured under 3, 5, 10 and 20% salinity were 3.03, 2.57, 2.50 and 2.43, respectively, indicating that the microbial diversity was decreased by the increased salinity. It is interesting to find that although consortium structure change a lot when cultured under 5 and 10% salinity, the degrading rate and the transcript of catabolic genes are not changed that much, suggesting CY-1 a strong functional redundancy at this salinity range. Various downstream pathways and catabolic genes redundancy were predicted to be the reason consortium CY-1 has such a high degree of functional redundancy (Chen et al. 2017; Dopheide et al. 2015). The community structure of a halophilic consortium reported by Guo et al. (2016) showed significant differences when cultured under 10 and 20% salt content. The same result was also concluded by Fang et al. (2016). In contrast to these results, the community structure of consortium CY-1 showed no significant differences when cultured at 10% and 20% salinity, indicating the community structure of consortium CY-1 was stable under hypersaline condition. However, as shown in Additional file 1: Figure S1, the transcription levels of all the catabolic genes were decreased with the increase of salinity, as well as the degradation rate of phenanthrene (Fig. 1), indicating the salinity can limit the microbial activity in halophilic consortium CY-1. Nonetheless, consortium CY-1 was identified as able to completely mineralize phenanthrene under 20% salinity as all catabolic gene were induced in the degradation process, indicating a potential use for PAHs remediation in hypersaline environments.

In summary, metabolic pathway of phenanthrene by halophilic consortium CY-1 was composed of a single upstream pathway and several downstream pathways, namely, the C12O, C23O, G12O and P34O pathway. The C23O pathway, mainly from *Marinobacter*, contributed more in the prophase of the PAH-degrading process; and the C12O pathway, mainly from *Halomonas* contributed more in the anaphase. Consortium CY-1 was able to mineralize phenanthrene in a wide range of salinities and maintain the community structure, especially in high salinity, demonstrating that consortium CY-1 has potential for the bioremediation of PAHs in hypersaline environments.

## Additional file

**Additional file 1: Table S1.** Accession numbers of the cloned catabolic gene sequences. **Figure S1.** Relative expression levels of genes encoding RHD, C12O, C23O, P34O, and G12O during phenanthrene degradation under different salinity. The transcript/gene ratios were calculated for the microcosm incubation. Three independent experiments were performed (error bars are not shown). A: Relative expression of genes encoding RHD; B: Relative expression of genes encoding C12O; C: Relative expression of genes encoding P34O; D: Relative expression of genes encoding C23O; E: Relative expression of genes encoding G12O.
