# Supplementary material for: Salinity effect on the metabolic pathway and microbial function in phenanthrene degradation by a halophilic consortium
Source: AMB Express. 2018 Apr 25;8:67. doi: 10.1186/s13568-018-0594-3 (PMC5918149; doi:10.1186/s13568-018-0594-3)
Supplement: Supplementary file 1 — Additional file 1: Table S1. Accession numbers of the cloned catabolic gene sequences. Figure S1. Relative expression levels of genes encoding RHD, C12O, C23O, P34O, and G12O during phenanthrene degradation under different salinity. The transcript/gene ratios were calculated for the microcosm incubation. Three independent experiments were performed (error bars are not shown). A: Relative expression of genes encoding RHD; B: Relative expression of genes encoding C12O; C: Relative expression of genes encoding P34O; D: Relative expression of genes encoding C23O; E: Relative expression of genes encoding G12O. [file 13568_2018_594_MOESM1_ESM.pdf]

AMB Express

Additional Material

Salinity Effect on the Metabolic Pathway and Microbial Function in Phenanthrene Degradation by a Halophilic Consortium

Chongyang Wang, Yong Huang, Zuotao Zhang, Hui Wang*

State Key Joint Laboratory of Environment Simulation and Pollution Control;
School of Environment, Tsinghua University, Beijing, 100084, China.

* Correspondence to Hui Wang: wanghui@tsinghua.edu.cn
Tel: 86-10-62772137; Fax: 86-10-62771472;

- Chongyang Wang, 13051971091@163.com;
- Yong Huang, hy791441103@yeah.net;
- Zuotao Zhang, zhangzuotaoaxy@163.com.

Table S1. Accession numbers of the cloned catabolic gene sequences.

number	protein	Sequences	Accession number
1	C12O	C12O in *Halomonas* LB1	MG827135
2	C12O	C12O in *Halomonas* LB12	MG827136
3	C12O	C12O in *Halomonas* G29	MG827137
4	C12O	C12O in *Halomonas* G31	MG827138
5	C12O	C12O in *Halomonas* G34	MG827139
6	C12O	C12O in *Martelella* N5	MG827140
7	C12O	C12O in *Chromohalobacter* LB10	MG827141
8	C12O	C12O in *Chromohalobacter* LB16	MG827142
9	C12O	OTU_{C12O} 1	MG827143
10	C12O	OTU_{C12O} 2	MG827144
11	C12O	OTU_{C12O} 3	MG827145
12	C12O	OTU_{C12O} 4	MG827146
13	C23O	C23O in *Marinobacter* N1	MG832594
14	C23O	C23O in *Marinobacter* N2	MG832595
15	C23O	OTU_{C23O} 1	MG832596
16	C23O	OTU_{C23O} 2	MG832597
17	P34O	P34O in *Martelella* N5	MG836548
18	P34O	P34O in *Halomonas* G29	MG836549
19	P34O	P34O in *Halomonas* G31	MG836550
20	P34O	OTU_{P34O} 1	MG832598
21	P34O	OTU_{P34O} 2	MG832599
22	P34O	OTU_{P34O} 3	MG832600
23	P34O	OTU_{P34O} 4	MG832601
24	G12O	G12O in *Halomonas* G29	MG836239
25	G12O	G12O in *Thalassospira* G41	MG836240
26	G12O	G12O in *Thalassospira* G42	MG836241
27	G12O	OTU_{G12O} 1	MG836242
28	G12O	OTU_{G12O} 2	MG836243
29	RHD	RHD in *Marinobacter* N1	MG836244
30	RHD	RHD in *Marinobacter* N2	MG836245
31	RHD	RHD in *Marinobacter* N4	MG836246
32	RHD	RHD in *Martelella* N5	MG836247
33	RHD	OTU_{RHD} 1	MG836248

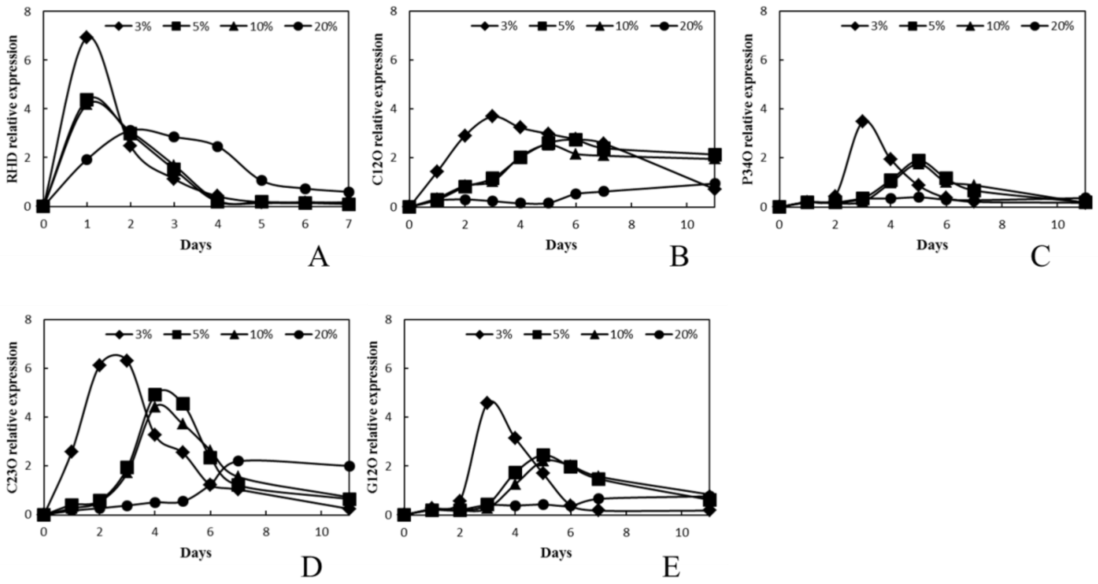

Figure S1. Relative expression levels of genes encoding RHD, C12O, C23O, P34O, and G12O during phenanthrene degradation under different salinity. The transcript/gene ratios were calculated for the microcosm incubation. Three independent experiments were performed (error bars are not shown). A: Relative expression of genes encoding RHD; B: Relative expression of genes encoding C12O; C: Relative expression of genes encoding P34O; D: Relative expression of genes encoding C23O; E: Relative expression of genes encoding G12O.
